# Online Personalized Normative Feedback to Foster Intention to Change and Help Seeking in Young Adults With Disordered Gambling and Trading Behaviors: Protocol for a Randomized Controlled Trial

**DOI:** 10.2196/73155

**Published:** 2026-02-23

**Authors:** Ainhoa Coloma-Carmona, José Luis Carballo, Fernando Miró-Llinares, Virtudes Pérez-Jover

**Affiliations:** 1 Center for Applied Psychology, Miguel Hernández University of Elche, Avenida de la Universidad s/n, Elche, 03202, Spain, +34 965222528; 2 Brief Intervention and Addictions Research Group (IBREA), Health Psychology Department, Miguel Hernández University of Elche, Elche, Spain; 3 Alicante Institute for Health and Biomedical Research (ISABIAL), Alicante, Spain; 4 CRÍMINA Research Center for the Study and Prevention of Crime, Miguel Hernández University of Elche, Elche, Spain

**Keywords:** young adults, gambling, trading, gambling disorder, trading disorder, brief intervention, normative feedback, clinical trial, study protocol

## Abstract

**Background:**

Despite the availability of treatment options, help-seeking rates among individuals with gambling problems remain low. To reach a broader population of those affected by disordered gambling, online and self-guided interventions have been developed. Personalized normative feedback (PNF) is one of the most widely used strategies for preventing gambling issues among young adults. However, most studies on PNF efficacy focus solely on its impact on the intensity and severity of gambling behavior, without exploring its potential to increase intention to change and help-seeking behaviors. Furthermore, there is a lack of studies assessing the efficacy of PNF in addressing emerging online gambling-like behaviors, such as betting within video games or excessive financial trading of high-risk assets (eg, cryptocurrencies), which have been linked to gambling disorder.

**Objective:**

This study aims to (1) quantify intention to change and the prevalence of help-seeking behaviors in young adults in Spain with disordered gambling or trading behaviors and (2) assess the efficacy of online PNF in increasing these behaviors.

**Methods:**

A randomized controlled trial using a Solomon 3-group design was conducted with a sample of emerging adults aged 18 to 34 years. The study included 3 assessments: a pretest, an immediate posttest, and a 12-week follow-up assessment. Participants were randomized into 1 of 3 conditions, with the intervention group receiving online PNF. The primary outcomes were intention to change and help-seeking behaviors. Secondary outcomes included gambling and trading behaviors (intensity, frequency, and severity) and their longitudinal trajectories. Individual, interpersonal, and contextual factors will be assessed to identify the profile of individuals most likely to benefit from this intervention.

**Results:**

The study was funded in December 2023 by the Spanish Ministry of Social Rights, Consumer Affairs and 2030 Agenda (SUBV23/00004) and approved by the Ethics Committee of the Miguel Hernández University. The first study assessment was conducted between December 2024 and January 2025. A total of 1889 people completed the eligibility assessment, of whom 1112 (58.9%) met the inclusion criteria (gambling or trading within the past 60 days) and were enrolled in the trial. The 12-week follow-up was finalized in March 2025, with 666 completing the final assessment (59.9% retention). Data collection has been completed, statistical analyses are ongoing, and primary results are expected to be published in March 2026.

**Conclusions:**

By examining motivational outcomes rather than behavioral change alone, this trial addresses a key gap in the literature on digital interventions for gambling-related harm. Findings are expected to inform the development of scalable prevention and early intervention strategies targeting gambling and gambling-like behaviors in young adults.

## Introduction

### Background

The digitalization of gambling markets has substantially transformed the gambling landscape, contributing to increased participation in online gambling and heightened exposure to gambling-related harms [1,2]. A recent systematic review and meta-analysis of representative samples across 68 countries found that 46.2% of adults gambled in the past 12 months, with 8.7% engaging in any risky gambling behavior, and that the prevalence of problem gambling was the highest among individuals participating in online casino or slot machine gambling (15.8%) [3]. In Spain, online gambling participation has tripled since 2013, and the prevalence of problem gambling among individuals who gamble online is also markedly higher than among those who gamble exclusively offline (18.4% and 4.3%, respectively) [4]. These patterns are particularly pronounced among young adults, an age group characterized by high exposure to digital environments and increased vulnerability to addictive behaviors [1,4-6].

Beyond traditional gambling products, digitalization has facilitated the expansion of gambling-like products that encourage monetary spending across multiple online contexts [2,6-9]. Within digital environments, gambling-like practices have been most extensively studied in the context of video games. In particular, loot boxes, mechanisms that involve spending real money on randomized virtual items or rewards, have been consistently associated with increased gambling severity and gambling-related harms among adolescents and young adults [10-14]. More recently, attention has also been drawn to skin gambling, which involves wagering of in-game items with real-world monetary value and has been suggested in recent studies to have stronger associations with gambling severity than loot boxes [6,15,16].

While younger adolescents (11‐14 years) represent the largest group of video game users, recent European data indicate that the greatest growth in gaming participation has occurred among individuals aged 25 to 34 years, underscoring the relevance of this age group for research on gambling-like digital spending [17].

In parallel, this same cohort has become increasingly active on online platforms and mobile apps that allow speculative trading of financial assets, such as cryptocurrencies and other high-risk instruments [7,18,19]. Unlike traditional long-term investing, speculative trading is characterized by short-term profit seeking, frequent transactions, and exposure to highly volatile markets, features that closely resemble those of gambling activities [9,20-23]. Moreover, trading platforms often incorporate *gamblified* design elements, such as frequent push notifications or visual cues emphasizing gains, which may reinforce risky decision-making and promote these activities as a form of entertainment [24-28].

An accumulating body of research indicates that speculative trading is associated with problem gambling, impulsivity, gambling-related cognitive distortions, and other established risk factors for gambling disorder [23,29-35]. Evidence also suggests a substantial overlap between gambling and trading behaviors. Previous studies have found that a large proportion of regular gamblers engage in financial trading, and that problem gambling severity is the strongest predictor of intense and maladaptive trading behaviors [32,34-36]. Importantly, rather than the use of specific financial instruments alone, the combination of speculative trading with multiple forms of gambling, particularly in online contexts, appears to identify subgroups with elevated risk profiles and greater gambling-related harm [23].

Despite this growing evidence base, gambling and trading behaviors are rarely examined jointly within intervention research, and trading is typically not systematically considered within prevention and treatment frameworks targeting gambling-related harms. A further challenge in addressing gambling-related problems is the extremely low rate of help seeking among affected individuals. A recent meta-analysis estimated that only 0.25% of people with gambling problems have ever sought formal help, a pattern observed consistently across types of gambling, countries, and treatment formats [37]. Available Spanish data on help seeking are largely derived from clinical samples already receiving specialized treatment and may therefore underestimate unmet need in the general population [38]. Commonly reported barriers to treatment include low motivation for change, stigma, and a preference for self-managing gambling-related difficulties [39-41]. These barriers are especially relevant for young adults, who are less likely to access traditional treatment services despite experiencing elevated levels of gambling-related risk [42-44].

To overcome these limitations, internet-based and self-guided interventions have been increasingly developed to reach individuals who are not in contact with specialized services [45-48]. Personalized normative feedback (PNF) is one of the most widely studied brief motivational interventions in this context, and it constitutes the main type of intervention used in the prevention of problem gambling among young adults [5]. As a core component of motivational interviewing approaches, PNF provides individualized feedback that compares an individual’s behavior with the observed behavior of a relevant reference group, such as peers, with the aim of highlighting discrepancies, fostering self-reflection, and supporting motivation to make informed decisions about problematic behavior [49].

Although meta-analytic evidence indicates that PNF produces only small reductions in gambling frequency and severity, these modest behavioral effects suggest that its primary utility may lie in enhancing motivation to change and promoting help seeking, rather than serving as a stand-alone behavioral intervention [50]. However, most existing PNF studies have focused on behavioral outcomes, with comparatively little attention to motivational end points such as intention to change or engagement with support resources. Moreover, PNF interventions have rarely been examined in relation to emerging gambling-like behaviors, including speculative trading or other forms of digital monetary spending. This gap is particularly relevant given that cryptocurrency trading is increasingly reported in gambling helpline contacts [51], underscoring the need for low-threshold interventions capable of addressing this broader risk landscape.

### Aims of the Project

The objective of this project was 2-fold: (1) to explore intention to change and help-seeking behavior among young adults with gambling and/or trading problems, given the heightened vulnerability of this age group [5,6], and (2) to evaluate the efficacy of an online PNF intervention in increasing intention to change and help-seeking behaviors in this population. As a secondary objective, the study examined whether exposure to PNF was associated with changes in gambling and trading behaviors (eg, frequency and intensity) over the follow-up period. Finally, the study compared the effects of PNF between individuals who engage in gambling or trading without meeting the criteria for a disorder (selective prevention) and those meeting the criteria for a gambling or trading disorder (indicated prevention).

## Methods

### Ethical Considerations

The study procedures were conducted in accordance with the Declaration of Helsinki and were approved by the Committee of Research and Ethics of the Miguel Hernández University of Elche (DPS.ACC.150523). All participants provided informed consent before participation. Data were collected through a secure web-based platform and were provided to the research team in anonymized form by the panel provider, with no access to personally identifiable information, thereby ensuring participant privacy and confidentiality. Participants received nonmonetary incentives in the form of redeemable points, in accordance with the panel provider’s standard procedures. The study protocol was prospectively registered in ClinicalTrials.gov (NCT06681103) on November 7, 2024, before participant enrollment.

### Study Design

This study was designed in accordance with SPIRIT (Standard Protocol Items: Recommendations for Interventional Trials) guidelines for clinical trial protocols (refer to the SPIRIT checklist in Checklist 1). A randomized controlled trial based on an adapted Solomon 3-group design was conducted, consisting of 2 control groups and 1 intervention condition. This design structure allows for the independent assessment of the immediate effects of the intervention on the primary motivational outcomes (ie, intention to change and help-seeking behaviors) while controlling for potential pretest sensitization (testing effects).

The study included a pretest, an immediate posttest (administered directly after the intervention or initial assessment where applicable), and a 12-week follow-up assessment to evaluate the maintenance of effects 3 months after the intervention. Although the classic Solomon design includes a fourth group that receives the intervention without a pretest [52], a pretest assessment is technically required in this study to generate the PNF for each participant. Accordingly, the adaptation proposed by Moreira et al [53] for studies applying PNF was adopted, omitting the intervention group with only a posttest.

Within the single intervention group (PNF), participants were randomly assigned to 1 of 2 predefined feedback assessment sequences (PNF-A vs PNF-B). Both subgroups received identical PNF content and completed the same primary and secondary outcome measures. The distinct feature between PNF-A and PNF-B lay in the timing of the postintervention assessment of gambling and trading behaviors (ie, frequency and intensity). In PNF-A, all posttest measures were completed after receiving PNF, whereas in PNF-B, participants completed posttest assessment of gambling and trading behaviors before receiving PNF and subsequently completed the posttest assessment of primary motivational outcomes after PNF.

This sequencing was intended to support secondary analyses examining potential reporting reactivity (eg, social desirability) in self-reported behavioral outcomes [54] and involved no alteration to the interventions’ delivery, content, or the assessment of the primary outcomes.

**Figure 1.**
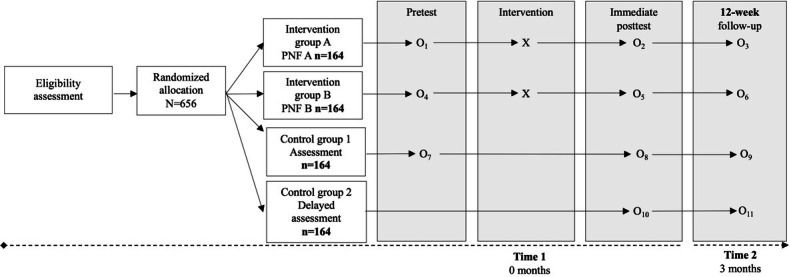
Flowchart of the adapted Solomon 3-group randomized controlled trial design, illustrating the assessment sequence across groups, including the intervention condition with randomized assessment sequencing (personalized normative feedback [PNF]-A and PNF-B). O1–O11 indicate assessment time points, and X denotes the intervention (PNF).

Accordingly, the study comprised three experimental conditions, with an additional within-intervention assessment sequencing: (1) a control group with pretest and posttest assessments (assessment group); (2) a control group with posttest assessment only (delayed assessment group); and (3) an intervention group receiving PNF, with pretest and posttest assessments and randomized assessment sequencing (PNF-A vs PNF-B; Figure 1).

### Participant Recruitment and Randomization

Recruitment targeted emerging adults aged 18 to 34 years. This specific age range was selected as it represents the demographic with the highest participation rates in the target behaviors, particularly in high-risk financial assets (eg, cryptocurrencies) and online gambling products [4,7]. In addition, this period of early adulthood is characterized by heightened exposure to digital environments and the adoption of novel gambling-like practices, which are less prevalent in older age groups and have been repeatedly examined in prior gambling research focusing on young and emerging adults [5,6,18,55].

Given the specific online nature of the behaviors assessed and the objectives of the project, recruitment was conducted in an online setting using an online panel managed by an independent Spanish agency specialized in ad hoc quantitative and qualitative research. Although nonprobability-based, this method was prioritized to effectively reach a geographically diverse group of young adults, as online panels offer the advantage of reaching a wider and more diverse group of young people compared with data collection in more specific contexts, such as university settings [56]. To contextualize sample representativeness, sociodemographic characteristics were benchmarked against a prior Spanish study using random digital sampling methods [7].

Participants’ eligibility was assessed in an initial screening, which included sociodemographic variables and measures of psychological distress, suicidal behavior, substance use, and gambling and trading participation in the past 60 days. Participants who met the inclusion criteria proceeded to the randomization stage. Eligible participants were those who (1) reported engaging in trading or gambling activities (land-based or online) at least once per month over the past 60 days; (2) were aged between 18 and 34 years; (3) resided in Spain; and (4) provided informed consent to participate in initial online assessment, posttest, and follow-up. Exclusion criteria included (1) currently undergoing psychological or psychiatric treatment at the time of assessment and (2) scoring 2 or more incorrect responses on the Oviedo Infrequency Scale-Revised (E Fonseca-Pedrero et al, unpublished document, 2008) indicating potentially random or dishonest responding.

Participant allocation to each condition was conducted via a computer-generated allocation algorithm integrated into the online survey platform. To ensure equal sizes across the 4 arms, the algorithm used a least-fill dynamic allocation method. This procedure automatically assigned each incoming participant to the experimental condition with the fewest enrolled participants at the time of entry, ensuring a balanced distribution of total participants across groups independent of baseline characteristics. In cases where 2 or more conditions had the same lowest number of participants, allocation was determined by simple random selection among those conditions.

### Sample Size Estimation

Given the lack of prior studies examining the impact of online PNF on intention to change and help seeking among individuals with gambling or trading problems, sample size estimation was based on previous trials evaluating PNF effects on gambling-related outcomes in young adults. Assuming an α error of 5%, an effect size f=0.15, and a correlation between baseline and follow-up measures of 0.5 [39,57], it was estimated that a total of 164 participants would be required to achieve a statistical power (1–β) of 90% for detecting differences within and between groups on a single outcome.

Given the evaluation of multiple outcomes, a minimum effective sample size of 328 was required. To account for potential attrition, the effective sample was adjusted using the following formula: final sample size=effective sample size/(1–loss to follow-up rate). Given the online panel setting, a conservative loss-to-follow-up rate of 50% was assumed before study launch. This adjustment yielded a target enrollment of 656 participants in total, corresponding to a minimum of 164 (25%) participants per randomized condition at baseline.

### Blinding

A double-blind design was used, wherein participants were unaware of their assigned experimental condition and were blinded to the study hypotheses regarding the efficacy of the intervention. In accordance with recommendations by Boutron et al [58], the outcome evaluation was conducted with researchers blinded to allocation to minimize the risk of detection bias.

### Intervention

The online PNF intervention provided participants with a brief report summarizing the results of their baseline assessment. This PNF included (1) an overview of the gambling and trading activities in which the participant engaged, including frequency of engagement and amounts spent; and (2) assessment scores on disordered trading (Trading Disorder Scale) and gambling (Problem Gambling Severity Index), along with interpretative information indicating levels of severity. Each result was accompanied by graphical representations, allowing participants to compare their scores with population-based norms from the Spanish demographic of the same sex and age group [3].

To mitigate the potential for increased gambling or trading behaviors in individuals whose current engagement levels are below the normative average, we incorporated the recommendations put forth by Newall et al [59] by presenting the normative data in conjunction with extreme values and relevant percentages.

### Eligibility Assessment

The eligibility assessment was conducted at baseline and included sociodemographic information such as sex, age, education, employment status, and monthly income. Psychological distress was assessed using the Kessler Psychological Distress Scale [60], which measures symptoms of nervousness, hopelessness, depression, and feelings of worthlessness over the past 4 weeks. A single item assessed current or past psychiatric or psychological treatment. Suicidal behavior was evaluated using the 5-item Paykel Suicide Scale [61] in its Spanish version [62], capturing death thoughts, ideation, and suicide attempts in the past year.

Substance use indicators were collected using items adapted from the Encuesta sobre Alcohol y otras Drogas en España (EDADES) survey [63]. Alcohol risk consumption was assessed using the 3-item version of the Alcohol Use Disorders Identification Test–Consumption [64]. Gambling and trading activities during the past 60 days were identified via ad hoc items based on the EDADES survey [63], capturing engagement in traditional gambling formats (eg, bingo, lotteries, and casino), emerging gambling-like activities (eg, buying loot boxes, e-sports betting, and skin betting), and speculative trading (eg, cryptocurrency trading, foreign exchange, and commodities).

### Primary Outcomes

Intention to change gambling or trading behavior was assessed through 3 ad hoc items. Participants answered a dichotomous item (yes or no) adapted to their chosen behaviors during the screening phase (eg, “In the last year, have you thought about reducing or quitting [financial asset trading/gambling]?”). Participants then reported the likelihood of engaging in gambling or trading over the next 12 months on a scale from 0% (very unlikely) to 100% (very likely). A third item asked participants to specify their actual plan regarding their behavior (not reducing or stopping, reducing or stopping in the following 6 months, and reducing or stopping in the following 30 days), with the latter 2 items administered at baseline, postintervention assessment, and follow-up.

Intention to seek treatment was also measured (at pretest, posttest, and at 12-week follow-up) using a likelihood item (0%‐100%). Help-seeking behaviors were additionally assessed at baseline using a question adapted from the study by Rodda et al [65] about prior help seeking (eg, “Have you ever sought professional help or advice, support from family or friends, or taken any self-directed actions to reduce your [gambling/trading] behavior?”). If answered affirmatively, participants completed an adaptation of the Help-Seeking Questionnaire [65], which includes items assessing self-directed, distance-based, and face-to-face help-seeking behaviors (eg, reading forums, browsing informational websites, and talking to a gambling counselor).

### Secondary Outcomes

Gambling and trading behaviors were assessed for frequency and intensity using items adapted from the EDADES survey [63] to capture gambling frequency and spending in both online and offline contexts. Trading behavior was assessed with items adapted from the study by Delfabbro et al [29], covering trading frequency, trading types, and frequency of monitoring financial markets. In the baseline and posttest assessments, these behaviors were measured retrospectively for the past year, while the follow-up covered the previous 3 months. Amateur or professional investment use was assessed with a dichotomous item (yes or no) regarding professional trading, as well as items on the use of broker platforms or apps.

Problem gambling was assessed using the 9-item Spanish version of the Problem Gambling Severity Index (PGSI) [66,67], categorizing participants into 4 groups: nonproblematic (PGSI=0), low-risk (PGSI=1‐2), moderate-risk (PGSI=3‐7), and problem gambling (PGSI≥8). Additionally, the Gambling Disorder Identification Test (GDIT) [68,69] was administered at follow-up as part of a planned validation process. The GDIT is a 14-item Likert scale instrument evaluating gambling behaviors, symptoms, and negative consequences. This scale allows for the classification of participants into the following categories: nongambling, recreational gambling, problem gambling, and pathological gambling (mild, moderate, or severe).

Disordered trading was measured with the Trading Disorder Scale [34], a validated 13-item instrument assessing problematic trading behaviors. The scale is based on research criteria proposed by Guglielmo et al [70], which are derived from selected *Diagnostic and Statistical Manual of Mental Disorders, Fifth Edition* (*DSM-5*) criteria for gambling disorder and internet gaming disorder [71] to conceptualize trading-related problems. This scale captures key aspects of behavioral addiction, such as tolerance, withdrawal, loss of control, craving, social impairment, and persistence despite negative consequences [34].

### Other Measures

Gambling- and trading-related harms were measured using the Short Gambling Harm Screen [72], a brief 10-item binary-response tool adapted to capture financial, emotional or psychological, and relational harms from gambling and trading. Perceived self-efficacy to resist gambling and trading urges was measured using an adaptation of the Brief Situational Confidence Questionnaire [73], which has been psychometrically validated for gambling-related behaviors in an adult general population sample [74]. This adapted version includes 2 additional items assessing confidence in resisting the urge to gamble when faced with situations involving financial pressure or the influence of alcohol or drugs, complementing the original 8 items focused on unpleasant emotions, physical discomfort, pleasant emotions, social pressure, and tests of control. Peer and family gambling and trading behaviors were assessed through ad hoc items. These items inquired about friends’ and family members’ involvement in financial trading and gambling. Finally, a 5-point Likert scale was used to evaluate satisfaction with and usefulness of the online PNF intervention (only in intervention groups).

### Data Analysis Plan

The data of the project will be made available upon reasonable request, supporting adherence to Open Science principles and ensuring transparency throughout the research process. Data will be analyzed using SPSS (version 28.0; IBM Corp), Mplus (version 8.7; Muthén & Muthén), and R (R Foundation for Statistical Computing). Descriptive analyses and group comparisons will be performed using chi-square tests for categorical variables and 2-tailed Student *t* test for independent samples, and ANOVA (including multivariate analysis of variance [MANOVA] and multivariate analysis of covariance [MANCOVA]) for continuous variables with a 95% confidence level. Data distribution will be tested with Kolmogorov-Smirnov and Shapiro-Wilk tests. If the assumption of normality is not met, nonparametric alternatives will be used. Effect sizes will be reported using appropriate statistics (eg, odds ratio, Cohen *d*, Rosenthal r, omega squared, or Cramer V). To control for type I error due to multiple comparisons, the Bonferroni correction will be applied. Results will be weighted by age, gender, and region.

To examine gambling and trading trajectories, primary longitudinal analyses will focus on generalized linear mixed models, which are preferred over traditional repeated-measures ANOVA due to their ability to handle missing data and accommodate nonnormal outcome distributions. Mixed latent class analyses will be used to identify latent subpopulations characterized by different patterns of gambling and trading intensity and severity over time. These analyses will be considered exploratory and contingent on data structure and model fit.

Mediation and moderation analyses will be conducted as secondary and exploratory analyses to examine potential mechanisms and conditional effects of the PNF intervention. These analyses will be implemented using the PROCESS macro for SPSS and structural equation modeling in Mplus to estimate direct and indirect effects, with continuous variables centered to reduce multicollinearity [75]. Bias-corrected 95% CIs will be generated from 10,000 bootstrap random samples.

During the evaluation of the PNF, observed variables will be controlled to ensure balanced baseline characteristics between control and experimental groups, thus confirming randomization integrity. Additionally, a comparative analysis will be conducted between participants who meet inclusion criteria (ie, engaging in gambling or trading activities in the past 60 days) and those who do not, assessing differences in sociodemographic variables, psychological distress, suicidal behavior, substance use, and gambling participation reported during eligibility assessment.

Questionnaires not previously validated in the Spanish population or for the constructs of interest will undergo psychometric evaluation, including item analysis, reliability, and validity testing. Item analysis will include item-test correlations, discarding items with discrimination indices below 0.30 [76]. Reliability analysis will be assessed using omega coefficients; a total omega ≥0.70 will be considered acceptable [77], while hierarchical omega will require a minimum of 0.50 per subscale [78]. Dimensionality and validity will be assessed through confirmatory factor analysis. Goodness-of-fit tests will be conducted, using the following indices and interpretations for the assessment of model fit: the comparative fit index, the relative goodness-of-fit index, the root mean square error, the Tucker-Lewis index, and the chi-square statistic corrected for degrees of freedom. A satisfactory model fit will be considered when the comparative fit index, Tucker-Lewis index, and relative goodness-of-fit index values are greater than 0.90; the root mean square error value is less than or equal to 0.05; and the chi-square statistic corrected for degrees of freedom values is close to 0 [79]. Measurement invariance will be tested across age, sex, and gambling or trading activity subgroups to confirm the stability of the factor structure across groups.

## Results

The study was funded in December 2023. Baseline data collection was conducted between December 2024 and January 2025. A total of 1889 individuals completed the eligibility assessment, of whom 1112 (58.9%) met the inclusion criteria and were enrolled in the trial. The 12-week follow-up was finalized in March 2025, with 666 (59.9% retention) participants completing the final assessment. As of January 2026, data collection has been completed, and statistical analyses are ongoing. Primary results are expected to be published in March 2026.

## Discussion

This study aims to address critical gaps in understanding emerging gambling and trading behaviors among young adults, a population that is increasingly involved in such activities, which have well-documented associations with psychological distress and gambling-related harm. Although generalizability may be influenced by the recruitment methodology, the online design of the study is particularly well suited to examining behaviors that occur primarily in digital contexts (eg, cryptocurrency trading and skin betting) [29,56,80] and enables timely and efficient assessment of motivational and behavioral outcomes over time [81].

To ensure data quality within this digital framework, sample characteristics were benchmarked against national reference data to contextualize representativeness [7]. Furthermore, to address limitations associated with self-reports, including social desirability bias, validated self-administered questionnaires and anonymized assessments were prioritized, and the Oviedo Infrequency Scale was included to detect and exclude random or dishonest responses. The use of multiple instruments for each measured variable will also allow for cross-verification, improving the reliability of the results. A key methodological strength is the use of an adapted Solomon 3-group experimental design, which enhances internal validity by allowing for the independent isolation of intervention effects from potential pretest sensitization.

This study represents a pioneering application of online PNF by extending it to the context of gambling-like practices, such as high-risk trading. To the best of our knowledge, this is one of the first randomized trials to assess the efficacy of PNF in increasing intention to change and help-seeking behaviors specifically within these emerging high-risk digital behaviors.

By examining the profiles of individuals who exhibit low motivation to seek help, the study also intends to provide insights that can contribute to the refinement of treatment and prevention strategies. However, several limitations should be acknowledged. First, longitudinal studies using online panels are susceptible to high attrition rates. To mitigate this, a conservative oversampling strategy (assuming 50% loss to follow-up) was used, and missing data will be handled via generalized linear mixed models. Second, as the sample is restricted to Spanish residents, cross-cultural generalizability may be limited. Finally, given the novelty of behaviors such as cryptocurrency trading, some measures were adapted ad hoc. Rigorous psychometric analyses are planned to validate these instruments as part of the study’s contributions.

Despite these limitations, findings may inform regulatory policies for trading platforms and gambling-like activities within video games to better protect vulnerable users. Excessive engagement in these activities has been associated with other addictive behaviors, such as disordered gambling and substance use, as well as emotional difficulties [82]. Furthermore, increased engagement in financial markets has been linked to rising suicide rates among both amateur and professional investors [70]. Consequently, the outcomes of the study are expected to advance public health by elucidating the psychological and social consequences of these converging behaviors and supporting the development of effective, scalable interventions aimed at reducing harm and facilitating help seeking in this population.

## Supplementary material

10.2196/73155 Checklist 1 SPIRIT 2025 checklist.

## Abbreviations

*DSM-5*: *Diagnostic and Statistical Manual of Mental Disorders, Fifth Edition*
EDADES: Encuesta sobre Alcohol y Drogas en España
GDIT: Gambling Disorder Identification Test
MANCOVA: multivariate analysis of covariance
MANOVA: multivariate analysis of variance
PGSI: Problem Gambling Severity Index
PNF: personalized normative feedback
PSS: Paykel Suicide Scale
SPIRIT: Standard Protocol Items: Recommendations for Interventional Trials
